# Salvianolic acid A promotes mitochondrial biogenesis and mitochondrial function in 3T3-L1 adipocytes through regulation of the AMPK-PGC1α signalling pathway

**DOI:** 10.1080/21623945.2022.2116790

**Published:** 2022-09-02

**Authors:** Jialin Sun, Ping Leng, Xiao Li, Qie Guo, Jun Zhao, Yu Liang, Xiaolei Zhang, Xue Yang, Jing Li

**Affiliations:** Department of Pharmacy, the Affiliated Hospital of Qingdao University, Qingdao, China

**Keywords:** Salvianolic acid A, 3T3-L1 adipocytes, mitochondrial biogenesis, mitochondrial function, AMPK, PGC-1α

## Abstract

Mitochondrial dysfunction is associated with insulin resistance and type 2 diabetes (T2DM). Decreased mitochondrial abundance and function were found in white adipose tissue (WAT) of T2DM patients. Therefore, promoting WAT mitochondrial biogenesis and improving adipocyte metabolism may be strategies to prevent and reverse T2DM. Salvianolic acid A (SAA) has been found to exert anti-diabetic and lipid disorder-improving effects. However whether SAA benefits mitochondrial biogenesis and function in adipose tissue is unclear. Here, we evaluated SAA’s effect on mitochondrial biogenesis and function in 3T3-L1 adipocytes and investigated its potential regulatory mechanism. Results showed that SAA treatment significantly promoted the transcription and expression of peroxisome proliferator-activated receptor γ coactivator- 1α (PGC-1α), nuclear respiratory factor 1 (NRF1) and mitochondrial transcription factor A (TFAM). Meanwhile, SAA treatment significantly promoted mitochondrial biogenesis by increasing mitochondrial DNA (mtDNA) quantity, mitochondrial mass, and expression of mitochondrial respiratory chain enzyme complexes III and complex IV. These enhancements were accompanied by enhanced phosphorylation of AMPK and ACC and were suppressed by Compound C, a specific AMPK inhibitor. Furthermore, SAA treatment improved adipocytes mitochondrial respiration and stimulated ATP generation. These findings indicate that SAA exerts a potential therapeutic capacity against adipocytes mitochondrial dysfunction in diabetes by activating the AMPK-PGC-1α pathway.

## Introduction

Mitochondria play a crucial role in many aspects of metabolism, and mitochondrial dysfunction is associated with insulin resistance and type 2 diabetes (T2DM) [1–3]. There is increasing evidence that, in patients with T2DM, the decline in mitochondrial metabolism and adenosine triphosphate (ATP) synthesis is consistent with a reduction in key factors regulating mitochondrial biogenesis [3–7]. Peroxisome proliferator-activated receptor γ coactivator- 1α (PGC-1α) is a master transcriptional regulator of mitochondrial biogenesis [8,9] and is affected by many transduction effectors such as adenosine monophosphate-activated protein kinase (AMPK), rapamycin (mTOR) and silent information regulator 1 (Sirt1) [8–10]. AMPK is known to be closely involved in cellular glucose metabolism and regulation of energy homoeostasis and is regarded as the potential therapeutic target for T2DM, obesity and metabolic syndrome [11–14].

White adipose tissue (WAT) is not only the main organ for energy storage, but also a very important endocrine organ. The hormones it secretes, including leptin, insulin resistin, adiponectin, etc., are important for regulating cellular energy homoeostasis [15].

Clinical studies have found a reduction in mitochondrial DNA (mtDNA) copy number in the adipose tissue of diabetic volunteers [16,17]. In addition, in patients with metabolic diseases such as obesity and diabetes, mitochondrial abundance and key genes related to mitochondrial function in WAT were significantly reduced [4,18,19]. Therefore, promoting WAT mitochondrial biogenesis and improving adipocyte metabolism may be strategies to prevent and reverse multiple diseases including cardiovascular disease, cancer, and insulin resistance.

Salvianolic acid A (SAA) ((2 R)-3-(3,4-dihydroxyphenyl)-2-[a-(3,4-dihydroxyphenyl) prop-2-enoyloxy] propanoic acid) is a polyphenol derivative extracted from the root of *Salvia miltiorrhiza*. It is known to possess extensive pharmacological activities including antioxidant [20], anti-inflammatory [21], and antiplatelet properties [22]. Furthermore, SAA exerts anti-diabetic and lipid disorder-improving effects, which are mainly manifested in reducing fasting blood glucose, fed blood glucose, total cholesterol, triglycerides, and low-density lipoprotein levels in diabetic rats [23,24]. At the same time, it can improve various diabetic complications such as diabetic nephropathy and diabetic peripheral neuropathy [25,26]. Notably, further studies showed that SAA regulates glucose metabolism by increasing ATP production, reducing mitochondrial membrane potential (MMP), and improving mitochondrial function through the Ca^2+^/ calmodulin-dependent protein kinase kinase β (CaMKKβ)/AMPK signalling pathway [23]. All these results suggest that SAA may be a promising candidate for the treatment of diabetes and its complications. However, to date, no studies have evaluated the effect of SAA on adipocytes, and it is unclear whether SAA acts directly on adipose tissue. Therefore, this study took 3T3-L1 adipocytes as the research object to evaluate the effect of SAA on mitochondrial function in 3T3-L1 adipocytes, and to investigate its potential regulatory mechanism.

## Materials and methods

### Cell Culture and Adipocyte Differentiation

3T3-L1 preadipocytes were obtained from the Procell Life Science&Technology Co., Ltd. (Wuhan, Hubei, China). After resuscitation, 3T3-L1 preadipocytes were cultured in Dulbecco’s modified Eagle’s medium (DMEM; #11965-092, Gibco, Carlsbad, CA, USA) supplemented with 10% calf serum (CS; #16010-159, Gibco, Carlsbad, CA, USA) and 1% penicillin-streptomycin (#10378-016, Gibco, Carlsbad, CA, USA) at 37°C in a humidified incubator containing 5% CO_2_. The differentiation process of the cells was performed as previously described [27]. Briefly, two days after cell confluency, cells were stimulated with DMEM containing 10% (v/v) foetal bovine serum (FBS; #10100-147, Gibco, Carlsbad, CA, USA), 10 *μ*g/ml insulin (#HY-P1156, MedChemExpress, Shanghai, China), 0.5 mM isobutylmethylxanthine (#HY-12318, MedChemExpress, Shanghai, China) and 1 *μ*M dexamethasone (#HY-14648, MedChemExpress, Shanghai, China) for 48 h to induce differentiation. Then, the culture medium was replaced with DMEM supplemented with 10% FBS and 10 *μ*g/ ml insulin. After 48 h, the medium was replaced with DMEM containing 10% FBS without insulin and changed every 2 days. Cells were used at 9 to 10 days after induction of differentiation, at which time 90% of cells exhibited an adipocyte phenotype.

### Drug Treatment

SAA (HPLC, 98%) was a gift from Prof. G.H. Du (Institute of Materia Medica, Chinese Academy of Medical Sciences, Beijing, China). Cells were incubated with SAA for 24 h to determine the effects of SAA in mitochondrial biogenesis. To clarify the possible involvement of AMPK, cells were stimulated with SAA in the presence or absence of the specific inhibitor Dorsomorphin (Compound C; #HY-13418A, MedChemExpress, Shanghai, China; 2.5 μM), and the results were compared with those of Acadesine (AICAR; #HY-13417, MedChemExpress, Shanghai, China; 0.5 mM), an activator of AMPK phosphorylation.

### Mitochondrial mass

The fluorescent probe MitoTracker™ Green FM (#M7514, Invitrogen, Carlsbad, CA, USA) was used to determine the mitochondrial mass of adipocytes. In the experiment, 3T3-L1 preadipocytes were cultured in 96-well clear bottom black culture solid plates. Adipocytes treated with SAA for 24 h were washed three times with serum-free minimum essential medium (MEM; #21090-055, Gibco, Carlsbad, CA, USA) pre-incubated at 37°C to completely remove the supernatant residue, and then incubated with 10 nM MitoTracker Green FM in serum-free MEM for 30 min at 37°C. After washing the cells three times with physiological saline, 100 μL of physiological saline was added to each well, and the fluorescence intensity of intracellular MitoTracker Green (Ex: 485 nm, Em: 515 nm) was detected by Multifunctional Microplate Reader SpectraMax M5 (Molecular Devices, LLC., San Jose, CA, USA).

### Electron microscopy observation

Adipocytes were prepared for electron microscopy after treatment with SAA for 24 h, performed as previously reported [28]. Briefly, cells were fixed overnight with 2.5% (v/v) glutaraldehyde in 0.1 M sodium phosphate buffer (pH = 7.3). After fixation with 2% (w/v) OsO_4_ in the same buffer, block staining was performed with 1% (w/v) uranyl acetate. Samples were subsequently dehydrated with a series of graded ethanol, washed with propylene oxide, embedded in Epon-812 epoxy resin, and finally cut into ultrathin sections. Six individual adipocytes treated with or without SAA were observed and measured by transmission electron microscopy (CM 120; Philips, Amsterdam, Netherlands).

For each individual adipocyte profile in the region, the number of mitochondria and total mitochondrial section area were determined. All electron microscopic photographs were analysed by observers blinded to treatments.

### Quantitative real-time polymerase chain reaction (qRT-PCR)

Total RNA was extracted from mature adipocytes with TRIzol reagent (#15596018, Invitrogen, Carlsbad, CA, USA), and the first-stand cDNA was synthesized using EasyScript® First-Strand cDNA Synthesis SuperMix (#AE301, TransGen Biotech Co., Ltd., Beijing, China), a cDNA synthesis kit. qRT-PCR was performed by CFX-96^TM^ real-time PCR system (Bio-Rad, USA) with commercial TransStart® Top Green qPCR SuperMix (#AQ131, TransGen Biotech Co., Ltd., Beijing, China). The relative gene expression levels were normalized to β-actin with the 2^−∆∆CT^ method. The primers used for amplification were synthesized by AuGCT Biotech Co., Ltd. (Beijing, China) and their sequences are listed in Table 1.

**Table 1.** Sequences of primers used for qRT-PCR.

Gene	Forward	Reverse
PGC-1α	5’-ATCTACTGCCTGGGGACCTT-3’	5’-ATGTGTCGCCTTCTTGCTCT-3’
NRF1	5’-CGCAGCACCTTTGGAGAA-3’	5’-CCCGACCTGTGGAATACTTG-3’
TFAM	5’-GGAATGTGGAGCGTGCTAAAA-3’	5’-TGCTGGAAAAACACTTCGGAATA-3’
β-actin	5’-ACGGCCAGGTCATCACTATTG-3’	5’-AGCCACCGATCCACACAGA-3’

### Mitochondrial DNA (mtDNA) assay

The mitochondrial number was quantified as previously described [29]. First, total DNA was extracted using DNA extraction kit (#DP304, Tiangen Biotech (Beijing) Co., Ltd., Beijing, China) following the manufacturer’s instruction. Then, the relative amounts of mtDNA were quantified by qRT- PCR with TransStart® Top Green qPCR SuperMix. The ratio of mtDNA to nuclear DNA (nDNA) was determined to quantify the mitochondrial content. 18S rRNA primers (forward: 5’-CATTCGAACGTCTGCCCTATC-3’; reverse: 5’-CCTGCTGCCTTCCTTGGA-3’) and mitochondrial D-loop primers (forward: 5’-AATCt-ACCATCCTCCGTG-3’; reverse: 5’-GACTAATGATTCTTCACCGT-3’) were used for nDNA and mtDNA target sequences, respectively.

### Western blot analysis

Western blot analysis was performed following the manufacturer’s instruction and previous studies [28]. After SAA treatment, 3T3-L1 adipocytes were washed twice with ice-cold PBS, and lysed in RIPA buffer. Homogenates were centrifuged at 12,000 × g for 10 min at 4°C. The protein concentration of the supernatant was determined by the BCA method. Denatured proteins were separated by sodium dodecyl sulphate-polyacrylamide (SDS-PAGE) gel electrophoresis, and transferred onto a polyvinylidene fluoride (PVDF) membrane. Membranes were blocked with 5% skim milk in Tris-buffered saline containing 0.1% Tween-20 (TBST) for 1 h at room temperature and incubated with primary antibodies at the recommended antibody dilutions at 4°C overnight, followed by incubation with horseradish peroxidase-conjugated secondary antibodies (goat anti-rabbit IgG, 1:3000, #E-AB-1003, Elabscience Biotech Co.,Ltd, Wuhan, Hubei, China; goat anti-mouse IgG, 1:3000, #E-AB-1001, Elabscience Biotech Co.,Ltd, Wuhan, Hubei, China) for 1 h at room temperature. The primary antibodies used in this study were anti-PGC-1α (1:5000, #66369-1-IG, Proteintech Group, Inc, Chicago, IL, USA), anti-nuclear respiratory factor-1 (anti-NRF1; 1:1000, #46743, Cell Signalling Technology, Danvers, Massachusetts, USA), anti-mitochondria transcription factor A (anti-TFAM; 1:3000, #22586-1-AP, Proteintech Group, Inc, Chicago, IL, USA), anti-phospho-(Thr 172)-AMPK (anti-p-AMPK; 1:1000, #2535, Cell Signalling Technology, Danvers, Massachusetts, USA), anti-total AMPK (anti-t-AMPK; 1:1000, #5831, Cell Signalling Technology, Danvers, Massachusetts, USA), anti-phospho-ACC (Ser79) (anti-p-ACC; 1:1000, #11818, Cell Signalling Technology, Danvers, Massachusetts, USA), anti-total ACC (anti-t-ACC; 1:1000, #3676, Cell Signalling Technology, Danvers, Massachusetts, USA), anti-cytochrome b (anti-CYTB; 1:600, #55090-1-AP, Proteintech Group, Inc, Chicago, IL, USA), anti-mitochondrial cytochrome c oxidase subunit I (anti-MTCO1; 1:2000, #ab14705, abcam, Cambridge, UK), anti-GAPDH (1:1000, #E-AB-20032, Elabscience Biotech Co.,Ltd, Wuhan, Hubei, China). Membranes were then washed and immunoreactive proteins were visualized using a chemiluminescent (ECL) assay kit (#E-IR-R307, Elabscience Biotech Co.,Ltd, Wuhan, Hubei, China) according to the manufacturer’s instructions. Protein bands were analysed using FluorChem Q gels image acquisition and analysis system (Bio-Techne, San Jose, CA, USA). The intensity of immunoreactive bands were quantitated by using ImageJ (NIH). The densities of the protein bands were normalized to GAPDH bands and quantified using ImageJ software (National Institute of Health, MD, USA).

### Extracellular oxygen consumption determination

Extracellular oxygen consumption rate of 3T3-L1 adipocytes after exposure to SAA for 24 h was measured using the extracellular O_2_ consumption assay kit (#ab197242, abcam, Cambridge, UK) following the manufacturer’s instructions. Fluorescence intensity was analysed by Multifunctional Microplate Reader SpectraMax M5 pre-heated at 37°C. Extracellular oxygen consumption signal was measured at 1.5 min intervals for 120 min at Ex/Em = 380/650 nm.

### ATP assay

Intracellular ATP levels in 3T3-L1 adipocytes were measured by a commercial ATP chemiluminescence assay kit (#E-BC-F002, Elabscience Biotech Co.,Ltd, Wuhan, Hubei, China) according to the manufacturer’s protocol. After 24 h incubation with SAA, cells were lysed and centrifuged at 10,000 g for 10 min at 4°C. The supernatant was collected for further assays. Chemiluminescent signals were collected at 636 nm by Multifunctional Microplate Reader SpectraMax M5.

### Statistical analysis

All data are representative of at least three independent experiments. Data are presented as mean ± standard error of the mean (SEM). Statistical analysis was performed by IBM SPSS Statistics Software v26.0 (IBM Co., Armonk, NY, USA). The normality of the variables was evaluated using the Shapiro-Wilk test, with homogeneity verified by the Levene test. Differences between two groups were compared by the Student’s t test or the Mann-Whitney U test, as appropriate. One-way ANOVA followed by the Turkey’s post hoc was used for multiple comparisons versus the control group. Results with a two-tailed adjusted *P* value < 0.05 were considered statistically significant.

## Results

### SAA stimulated transcriptional activity and protein expression of PGC-1α

**Figure 1.**
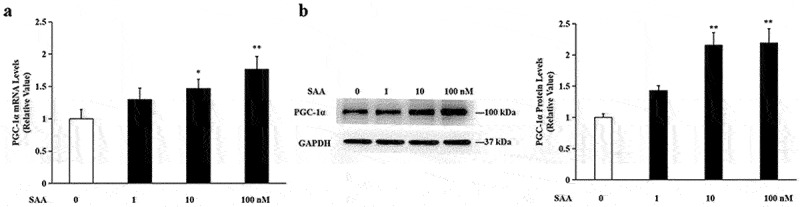
**SAA promotes the expression of PGC-1α in 3T3-L1 adipocytes cells**. 3T3-L1 cells were incubated with various concentrations of SAA for 24 h. (a) mRNA levels of PGC-1α were determined by qRT-PCR analysis; (b) Protein levels of PGC-1α were determined by Western blot analysis. Data are expressed as mean ± SEM of three independent measurements. *P < 0.05, **P < 0.01 vs. non-treated group.

PGC-1α is a key factor driving mitochondrial biosynthesis, and it also plays an important stimulatory role in thermogenesis and fatty acid oxidation in muscle and adipose tissue [29–31]. Treatment of adipocytes with 10^−9^-10^−7^ mol/L of SAA for 24 h resulted in a dose-dependent and sustained increase in the messenger RNA (mRNA) levels (Figure 1a) and protein levels (Figure 1b) of PGC-1α.

### SAA up-regulated genes involved in mitochondrial biogenesis

**Figure 2.**
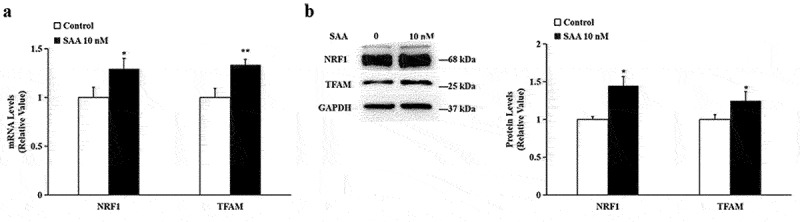
**SAA promotes the expression of NRF1 and TFAM in 3T3-L1 adipocytes cells**. 3T3-L1 cells were incubated with 10 nM SAA for 24 h. (a) mRNA levels of NRF1 and TFAM were determined by qRT-PCR analysis; (b) Protein levels of NRF1 and TFAM were determined by Western blot analysis. Data are expressed as mean ± SEM of three independent measurements. *P < 0.05, **P < 0.01 vs. non-treated group.

The PGC-1α-NRF1-TFAM pathway plays a key role in regulating mitochondrial biogenesis. Therefore, we further examined the effects of SAA on the gene expression of NRF1 and TFAM, two important downstream target genes of PGC-1α. Treatment of SAA at 10^−8^ mol/L resulted in a significant increase of NRF1 and TFAM in 3T3-L1 cells at both mRNA levels (Figure 2a) and protein levels (Figure 2b).

### SAA promotes mitochondrial biogenesis

**Figure 3.**
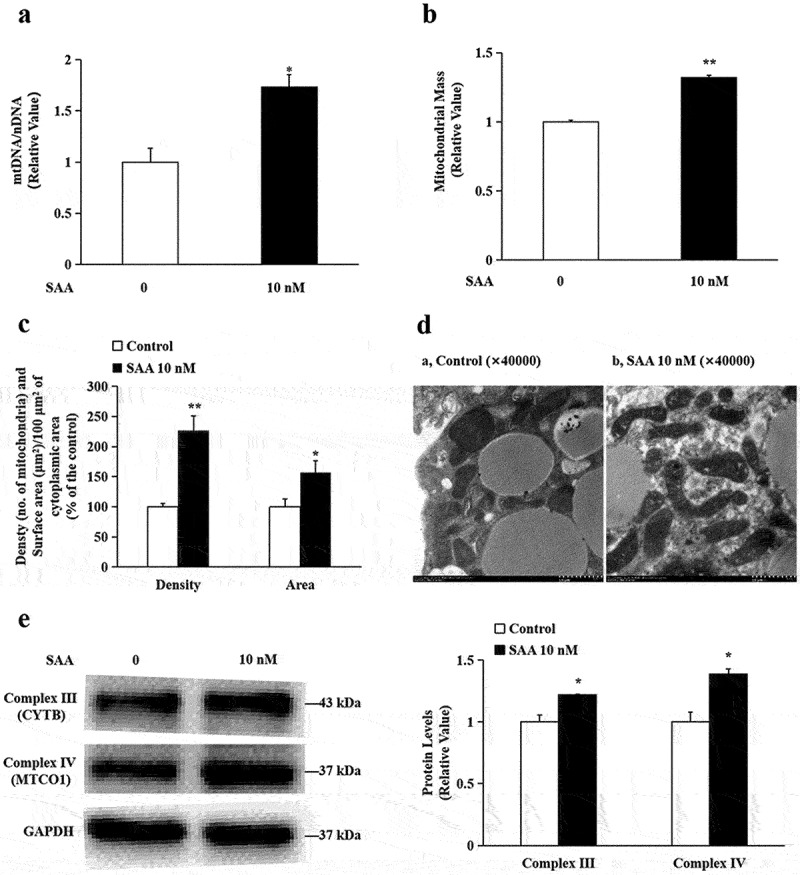
**SAA promotes mitochondrial biogenesis in 3T3-L1 adipocytes cells**. 3T3-L1 cells were incubated with 10 nM SAA for 24 h. (a) Mitochondrial-to-nuclear DNA (mtDNA/nDNA) was determined by qRT-PCR analysis. Data are expressed as mean ± SEM of three independent measurements; (b) Mitochondrial mass was determined by staining with MitoTracker Green dye. Data are expressed as mean ± SEM of three independent measurements; (c) Morphometric analysis of surface area and density of mitochondria under the electron microscope. Data are expressed as mean ± SEM of data from six cells; (d) Representative illustrations of mitochondrial profiles under the electron microscope (magnification, ×40,000); (e) Protein levels of oxidative phosphorylation complex III (CYTB) and complex IV (MTCO1) were determined by Western blot analysis. Data are expressed as mean ± SEM of three independent measurements. *P < 0.05, **P < 0.01 vs. non-treated group.

The D-loop is the primary site for transcription initiation on the heavy and light chains of mitochondrial DNA (mtDNA). TFAM has been reported to play a critical role in activating the promoter and initiating D-loop transcription of mtDNA. As SAA stimulates mRNA expression of TFAM, it is expected that mtDNA copy number should be increased. To verify whether SAA treatment increases mitochondrial mass, we measured mtDNA contents. As shown in Figure 3a, SAA treatment at 10^−8^ mol/L resulted in a significant increase in the ratio of mitochondrial D-loop/18s rRNA.

We then used MitoTracker Green FM, a specific fluorescence dye which stains mitochondria in a mass-dependent fashion, to label and quantify mitochondria in 3T3-L1 cells. Results showed that, 10^−8^ mol/L SAA treatment resulted in a significant increase in fluorescence intensity, suggesting an increase in mitochondrial mass (Figure 3b).

Mitochondrial morphology was also examined under the electron microscope. Quantitative analysis (six cells were analysed) demonstrated that the treatment with SAA at 10^−8^ mol/L for 24 h significantly increased the total mitochondrial section area to 156.5 ± 20.3% of control, and also increased mitochondrial numbers to 226.7 ± 23.9% of control (both *P* < 0.05). The results were shown in Figure 3c, and, representative control adipocytes and 10^−8^ mol/L SAA-treated adipocytes were shown in Figure 3d.

### SAA increased the protein expression of mitochondrial respiratory chain enzyme complexes

Mitochondrial respiratory chain complexes are the main functional proteins of oxygen consumption and ATP synthesis in the body, and their coding is jointly regulated by nuclear DNA and mtDNA. Here, we measured the expression of two mitochondrial proteins, Complex III and Complex V. Western blot analysis in Figure 3e indicated that SAA treatment significantly increased the expression of the Complex III subunit 3 (CYTB) and Complex IV subunit 1 (MTCO1).

### SAA promoted mitochondrial biogenesis mediated by the AMPK pathway

**Figure 4.**
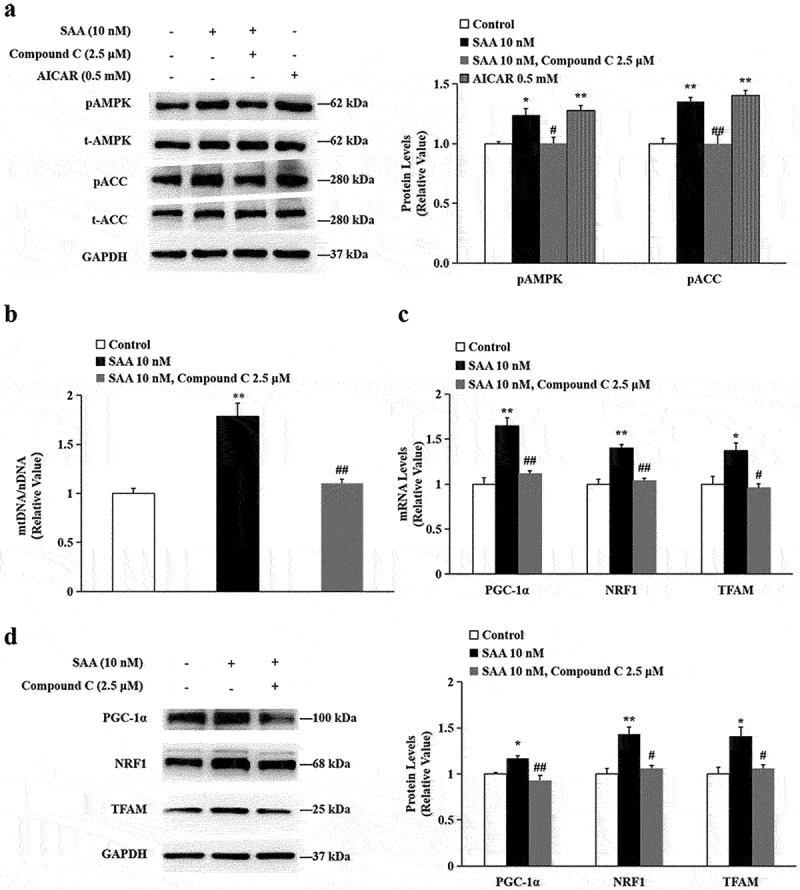
**The effects of SAA on PGC-1α are mediated by the AMPK pathway**. 3T3-L1 cells were incubated with 10 nM SAA for 30 min with or without the specific AMPK inhibitor Compound C (2.5 μM), or incubated with 0.5 mM AMPK activator AICAR for 30 min. (a) Phosphorylated levels of AMPK and ACC were determined by Western blot analysis.The ratios of p-AMPK:t-AMPK and p-ACC:t-ACC were calculated and compared; (b) mtDNA/nDNA was determined by qRT-PCR analysis; (c) mRNA levels of PGC-1α, NRF1 and TFAM were determined by qRT-PCR analysis; (d) Protein levels of PGC-1α, NRF1 and TFAM were determined by Western blot analysis. Data are expressed as mean ± SEM of three independent measurements. *P < 0.05, **P < 0.01 vs. non-treated group; ^#^*P* < 0.05, ^##^*P* < 0.01 vs. SAA treated group.

AMPK is a master regulator of mitochondrial biogenesis through activation of PGC-1α. To explore the mechanisms underlying SAA-induced PGC-1αexpression, we investigated the phosphorylation of AMPK (Thr 172) and acetyl-CoA carboxylase (ACC) in adipocytes after SAA treatment. As expected, SAA treatment significantly increased the phosphorylation of AMPK and ACC, similar to the AMPK activator AICAR. And the enhancement effect were abolished by compound C (Figure 4a), a specific AMPK inhibitor. In addition, SAA (10^−8^ mol/L)-induced increase in mtDNA encoding (Figure 4b), gene (Figure 4c) and protein expressions (Figure 4d) of PGC-1α, NRF1, and TFAM were all abolished by compound C treatment, confirming the involvement of the AMPK pathway.

### SAA improved mitochondrial respiration and stimulated ATP generation

**Figure 5.**
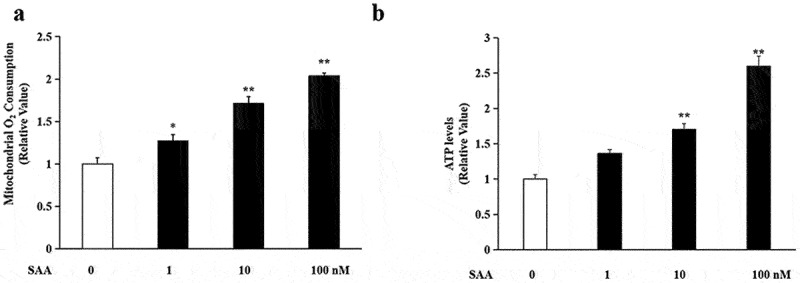
**Protective effects of SAA on the mitochondrial respiration and energy metabolism of 3T3-L1 adipocytes cells**. 3T3-L1 cells were incubated with various concentrations of SAA for 24 h. (a) O_2_ consumption rates. Data are expressed as mean ± SEM of five independent measurements; (b) ATP production. Data are expressed as mean ± SEM of three independent measurements. *P < 0.05, **P < 0.01 vs. non-treated group.

It has been proposed that mitochondrial biogenesis activation could lead to a functional gain of mitochondria. To access whether SAA treatment could enhance mitochondrial function in 3T3-L1 cells, mitochondrial oxygen consumption was measured and mitochondrial respiratory rate was calculated. As shown in Figure 5a, 10^−9^-10^−7^ M SAA treatment for 24 h significantly increased oxygen consumption and the respiratory rate in a dose-dependent manner (*P* < 0.01). Mitochondria are the major sites of ATP production in cells. The measurement of ATP levels showed that SAA (10^−9^, 10^−8^ and 10^−7^ M) stimulation for 24 h increased intracellular ATP levels by 36%, 70% and 160%, respectively, compared to the control group (*P* < 0.01) (Figure 5b). The results suggested that SAA had a positive effect on the enhancement of mitochondrial respiration and energy metabolism in adipocytes.

## Discussion

T2DM has become one of the major public health problems worldwide. Metabolic disorders is an important pathological basis leading to insulin resistance in the body. Adipocytes play a central role in maintaining lipid homoeostasis and energy balance [4,15]. Abnormal adipocyte lipolysis has been implicated in T2DM, and targeting adipocyte lipolysis has therapeutic implications. Under the induction of specific inducers, 3T3-L1 preadipocytes can proliferate and differentiate into adipocytes, so they are widely used in studies related to glycolipid metabolism. As the “energy factories” of cells, mitochondria are the main site for glycolipid metabolism and oxygen consumption, which provide more than 95% of the ATP required for cell metabolism [30]. Mitochondrial dysfunction has been found in clinical cases of a large number of metabolic diseases, including T2DM. Therefore, improving mitochondrial function of adipocytes may be strategy for the treatment of metabolic diseases.

SAA is a water-soluble extract derived from *Salvia Miltiorrhiza* Bunge. Previous studies have found that SAA can improve the disrupted lipid profiles in diabetic rats [24], and at the same time, ameliorate mitochondrial respiratory dysfunction in multiple organs such as the heart, liver, and skeletal muscle [23,31,32]. Studies suggested that SAA might exhibit antidiabetic effects by targeting mitochondria to improve lipid metabolism. Therefore in this study, we sought to explore its effect on mitochondrial function in 3T3-L1 adipocytes.

Our data showed that stimulation of 3T3-L1 adipocytes with 10^−9^-10^−7^ M SAA exhibited a dose-dependent induction on PGC-1α. Meanwhile, 10^−8^ M SAA treatment induced NRF1 and TFAM expression in adipocytes. PGC-1α is known to regulate the expression of NRF1, which further regulates the expression of TFAM [33]. After transcribed in the cytoplasm, TFAM is transported to mitochondria and acts as a key regulator of mitochondrial transcription and replication [34,35]. The PGC-1α-NRF1-TFAM network is closely related to the regulation of mitochondrial biogenesis in cells [8,9,33,34]. Based on the induction of these key mitochondrial regulators, SAA is expected to exert biological effects on adipocyte mitochondrial biogenesis. Then, we further assessed its effect on mitochondrial mass and function.

MtDNA contents and mitochondrial mass levels were increased in SAA-treated 3T3-L1 adipocytes compared to untreated controls. Meanwhile, SAA treatment enhanced the levels of two mitochondrial proteins, complex III and complex IV, suggesting that increased mitochondrial biogenesis signalling leads to enhanced mitochondrial protein synthesis. In addition, our findings showed that SAA treatment significantly increased oxygen consumption and ATP production, thereby improving mitochondrial respiration. However, the magnitude of the enhancement of mitochondrial respiration is similar to that of mtDNA, so whether this enhancement is due to changes in the number of mitochondria or an increase in the function of each mitochondria remains to be further investigated.

AMPK has been shown to be an upstream regulator of PGC-1α [8,9,33]. Therefore, we hypothesized in this study that SAA might induce PGC-1α expression in adipocytes by activating the phosphorylation of AMPK. Our data indicated that SAA had an activating effect on the phosphorylation of AMPK and ACC. The addition of the AMPK specific inhibitor Compound C abolished the induction of PGC-1α, NRF1, and TFAM by SAA and its influence on the mtDNA/nDNA ratio, suggesting the involvement of AMPK.

WAT swelling, reduced adipose tissue browning, and reduced brown adipose tissue activity are important pathological basis of metabolic syndromes such as obesity and T2DM. Our study focused on the effects of SAA on mitochondrial biogenesis and energy metabolism in white adipocytes and the related mechanisms, but whether it can promote adipose tissue browning and brown adipose tissue activity has not been thoroughly investigated. Excitingly, Lai et al. have conducted in-depth studies on the browning of WAT under the action of SAA at the animal and cellular levels. Their study found that SAA can promote the expression of brown adipose tissue marker protein uncoupling protein 1 (UCP-1), thereby promoting WAT browning; and increase the gene expression of PGC-1α, indicating that it has the effect of promoting mitochondrial biogenesis in adipocytes. They also demonstrated that AMPK phosphorylation is mechanistically involved in SAA-induced UCP-1 upregulation. The above results also support our findings.

Compared with WAT, brown adipose tissue has higher mitochondrial abundance and greater metabolic activity and capacity, but whether SAA can play a similar role in the regulation of energy metabolism in brown adipocytes has not yet been reported. In the study of Lai et al., the expression of UCP-1 in brown adipose tissue was analysed under the action of SAA, and no statistical difference was found. As a mitochondrial inner membrane protein, does the lack of differential expression of UCP-1 imply that SAA has no effect on mitochondrial biogenesis in brown adipose tissue? Not sure yet.

In conclusion, the present study provides direct experiment evidence that SAA exerts positive effects on adipocytes mitochondrial biogenesis and function. The molecular mechanism of SAA partly involves the regulation of AMPK and its downstream ACC and PGC-1α-NRF1-TFAM pathway. Our results indicate that SAA is a potential agent for the treatment of T2DM. Future experiments should be performed to systematically investigate the effect of SAA on mitochondrial biogenesis and function in brown adipose tissue and to confirm its effects in animals.

## Data Availability

Data sets used during the current study are available from the corresponding authors upon reasonable request (https://fairsharing.org/users/6944).

## Declarations

The study was conducted in accordance with the World Medical Association Declaration of Helsinki and was approved by the Ethics Committee of the Affiliated Hospital of Qingdao University.
